# Caloric; Its Medicinal, Chemical, and Vital Agencies on the Phenomena of Nature

**Published:** 1845-09

**Authors:** 


*Caloric; its Medicinal, Chemical, and Vital agencies on the Phenomena of Nature.* By SAMUEL L. METCALFE, M. D., of Transylvania University. 8vo. 2 vols. London, 1843.

